# Antimicrobial resistance and vaccine-preventable infections: concurrent health security challenges after the double earthquake in Venezuela

**DOI:** 10.17843/rpmesp.2026.433.17138

**Published:** 2026-09-01

**Authors:** María Araque

**Affiliations:** 1 Laboratorio de Microbiología Molecular, Facultad de Farmacia y Bioanálisis, Universidad de Los Andes, Mérida, Venezuela. Universidad de Los Andes Laboratorio de Microbiología Molecular Facultad de Farmacia y Bioanálisis Universidad de Los Andes Mérida Venezuela

The double earthquake that struck central-northern Venezuela last June marked a turning point in the country’s public health memory. The disaster had a devastating impact, severely compromising critical infrastructure and the functionality of the healthcare delivery network. According to estimates by the United Nations Children’s Fund (UNICEF), approximately 6.76 million people were potentially affected, resulting in the mass displacement of thousands of families to overcrowded shelters ^1^. In addition to the structural damage, the catastrophe highlighted the complex interplay between the historical vulnerabilities of Venezuela’s healthcare system and the emergence of global biological threats. In this context, the convergence of an overwhelmed hospital network, disrupted access to essential healthcare services, and inadequate conditions in improvised shelters creates an environment suitable for the empirical and indiscriminate use of antimicrobials and for the imminent reemergence of outbreaks of vaccine-preventable diseases.

The assessment of the risk of post-disaster infectious diseases outbreaks should be interpreted within the context of a pre-existing humanitarian crisis in Venezuela, which had already severely weakened the national immunization program. As a critical background factor, the national healthcare system was experiencing significant deterioration due to financial and operational constraints driven by socioeconomic and political factors ^2^. The cessation, since 2018, of essential vaccines against pneumococcal disease (PCV) and rotavirus (RV) vaccination has led to accumulate a cohort of unprotected children. In addition to this baseline vulnerability, the earthquake disrupted electricity and drinking water supplies, disrupting the cold chain required for the conservation of available biological products ^1^^-^^3^. Vaccination coverage was already low before the disaster, with only 51.1% of children receiving the second dose of measles-containing vaccine (MCV2) and 62.1% receiving the third dose of the pentavalent vaccine (DPT3) ^3^^,^^4^. These coverage gaps entail an imminent risk of active transmission and outbreaks of diseases such as measles, bacterial pneumonia, and acute diarrheal diseases, particularly in temporary shelters, where overcrowding accelerates the spread of these infectious conditions ^4^^,^^5^. Accordingly, various scientific and public health stakeholders have emphasized the need to strengthen vaccination as an integral component of the emergency response, particularly among children and displaced populations ^1^^,^^3^^-^^5^.

According to World Health Organization (WHO) guidelines, the response should not focus on mass vaccination campaigns, but rather on promptly restoring the routine immunization schedules that were already in place ^6^. In addition, specific high-risk groups should be immunized as a preventive component of the immediate response. This technical priority prevents that pre-existing gaps in immunization coverage trigger outbreaks of vaccine-preventable diseases in overcrowded settings ^4^^-^^6^.

At the same time, natural disasters can act as indirect catalysts for the emergence and spread of antimicrobial resistance (AMR), a phenomenon that is currently being investigated in the context of recent seismic events, including those in Türkiye and Syria in 2023 ^7^. In disaster settings, high clinical workload, diagnostic uncertainty, and clinical concern may promote the empirical and indiscriminate use of broad-spectrum antimicrobial drugs. Such indiscriminate empiricism leads to inappropriate antibiotic use, exerting significant selective pressure on patients’ microbiota, leading to dysbiosis. Moreover, recurring factors such as treatment interruptions, unplanned changes in therapeutic regimens, and the lack of systematic monitoring of prescriptions support the hypothesis that the emergency response itself may become an amplifier of the biological crisis in both hospital and community settings ^7^^,^^8^.

Before the double earthquake, Venezuela was already experiencing a substantial burden of antimicrobial resistance (AMR), lacking a comprehensive epidemiological surveillance and a national action plan, with a fragmented healthcare system and an uneven distribution of diagnostic and therapeutic resources ^3^^,^^9^. The added strain of the seismic emergency on these pre-existing vulnerabilities increases the risk of healthcare-associated infections (HAIs) spreading within overburdened healthcare facilities.

In this context of crisis, clinical microbiology and infectious diseases medicine play strategic roles. Clinical microbiology laboratories are essential for identifying pathogens responsible for traumatic wound infections, pneumonia, and bloodstream infections. They also provide antimicrobial susceptibility profiles that guide initial empiric therapy and subsequent de-escalation or adjustment of treatment ^1^^,^^3^^,^^7^^,^^8^. Infectious diseases specialists, in turn, can develop protocols tailored to resource-limited settings and provide guidance on antimicrobial stewardship. International experience from recent earthquakes has shown that integrating infectious diseases expertise, clinical microbiology, and public health surveillance into disaster response committees is essential ^7^^-^^9^. This articulation enables the optimization of diagnostic processes, the containment of infections caused by multidrug-resistant organisms, a more rational use of antibiotics, and the implementation of infection prevention and control measures adapted to the realities of the emergency setting.

Facing such a scenario, it is necessary to move beyond merely documenting vulnerabilities towards the development of an agenda for effective action built around three strategic pillars. First of all, disaster response plans should explicitly include measures to contain AMR and prevent HAIs. This strategic pillar is supported by epidemiological evidence showing that major earthquakes can trigger a “second wave” of health emergencies, characterized by complex crush injuries which are frequently heavily contaminated and require repeated surgical debridement and prolonged stays in intensive care units (ICUs). Under the intense clinical pressure seen during the response phase, these clinical conditions can accelerate the selection and dissemination of multidrug-resistant Gram-negative bacilli, such as *Acinetobacter baumannii, Pseudomonas aeruginosa*, and extended-spectrum β-lactamase (ESBL)- and/or carbapenemase-producing Enterobacterales, substantially increasing the rates of amputation, organ failure, and post-traumatic mortality ^7^^-^^9^. Given the setting of collapsed infrastructure, microbiological diagnosis should be prioritized through strategies that promote technological autonomy. Technologies such as telemicrobiology and rapid diagnostic tests, including molecular point-of-care testing (POCT), can enable the identification of pathogens and resistance biomarkers within the first few hours ^9^^,^^10^. This approach makes it possible to move away from blind empiricism, facilitate targeted therapy with narrow-spectrum antibiotics, and preserve the clinical utility of last-resort antimicrobials by limiting the selection and dissemination of multidrug-resistant pathogens.

Second, it is essential to implement a minimum set of indicators for monitoring antimicrobial resistance (AMR) and antimicrobial consumption in the post-disaster setting. Prescription monitoring in affected areas should be conducted in accordance with Pan American Health Organization (PAHO) guidelines and structured around the World Health Organization (WHO) AWaRe classification (Access, Watch, Reserve) framework. This tool enables the rapid categorization and auditing of available therapeutic resources, prioritizing the prescription of antibiotics in the Access group while strictly regulating the empirical use of Watch and Reserve agents by medical response teams ^11^. In this way, simplified data collection serves as an early warning mechanism for deviations from local clinical protocols and provides a standardized, comparable metric for assessing the quality of healthcare response during crisis situations.

Third, as a cornerstone of health security, the emergency response should consolidate a dedicated immunization axis to position vaccination as an immediate operational priority rather than a deferred intervention ^4^^,^^6^. Interventions should be structured across multiple dimensions. From a clinical and service-delivery perspective of immunizations, this includes a rapid assessment of vaccination status and the implementation of immunization strategies integrated into primary care and , with priority given to children requiring protection against measles through the measles, (MMR vaccine), as well as against diphtheria and pertussis through the pentavalent vaccine. Influenza and COVID-19 vaccination should also be prioritized among high-risk groups ^3^^,^^4^. From an infrastructure-support perspective, it is essential to ensure the sustained restoration of routine immunization services, including reliable supplies of vaccines and other biological products, maintenance of the cold chain through emergency generators, and strengthened technical training for healthcare personnel ^3^. Finally, from a preventive epidemiological perspective, vaccination strategies should be adapted to post-disaster risks, including anti-tetanus prophylaxis for debris-related wounds and targeted hepatitis A vaccination in response to persistent inadequate sanitation conditions in shelters, closely integrated with active syndromic surveillance ^3^^,^^4^.

The devastation caused by the double earthquake in Venezuela serves as a reminder that public health is built not only on managing day-to-day stability, but also on the capacity of health systems to anticipate, withstand, and learn from crises. This experience extends beyond the national context and should serve as a reference for all of Latin America, with particular emphasis on the Andean region. This referential framework provides an opportunity to build on successful interventions and critically assess operational shortcomings, transforming lessons learned into an opportunity to strengthen institutional resilience in the face of future emergencies. The region shares a high degree of geophysical vulnerability due to complex active tectonic systems, resulting in recurrent exposure to major seismic disasters. This structural vulnerability is compounded by extreme climate-related susceptibility to meteorological phenomena such as El Niño, whose alterations in precipitation and temperature patterns promote vector proliferation and exacerbate outbreaks in settings characterized by overcrowding and social disadvantage. When such disasters affect already weakened health systems—characterized by suboptimal immunization coverage and the collapse of water, sanitation, and hygiene (WASH) services—the health consequences extend well beyond the immediate acute trauma. Safeguarding access to vaccines and strengthening population immunity during crisis situations constitute essential frontline operational measures for containing preventable outbreaks. At the same time, at this critical intersection, antimicrobial resistance (AMR) emerges as a superimposed and largely silent second biological crisis. Consequently, disaster management policies across the region must necessarily include AMR containment, access to and strengthening of microbiological diagnosis, and the preservation of immunization programs as fundamental pillars of collective health security.
