# Supplementary material for: Antimicrobial resistance and vaccine-preventable infections: concurrent health security challenges after the double earthquake in Venezuela
Source: Rev Peru Med Exp Salud Publica. 2026 Sep 1;43(3):298–300. doi: 10.17843/rpmesp.2026.433.17138 (PMC13718567; doi:10.17843/rpmesp.2026.433.17138)
Supplement: Translation

# Translation: Resistencia antimicrobiana e infecciones inmunoprevenibles: desafíos concurrentes de seguridad sanitaria tras el doble terremoto en Venezuela

**Authors:** María Araque

**Affiliations:** 1 Laboratorio de Microbiología Molecular, Facultad de Farmacia y Bioanálisis, Universidad de Los Andes, Merida, Venezuela.

El doble terremoto registrado en el centro-norte de Venezuela el pasado mes de junio marcó un punto de inflexión en la memoria sanitaria del país. El desastre causó un impacto devastador, comprometiendo gravemente la infraestructura crítica y la funcionalidad de la red de servicios de salud. De acuerdo con estimaciones del Fondo de las Naciones Unidas para la Infancia (UNICEF), aproximadamente 6,76 millones de personas se vieron potencialmente afectadas, provocando el desplazamiento masivo de miles de familias hacia refugios en condiciones de hacinamiento ^1^. Además de los daños estructurales, esta catástrofe puso de manifiesto la compleja interacción entre las fragilidades históricas del sistema sanitario venezolano y el surgimiento de amenazas biológicas globales. En este contexto, la confluencia de una red hospitalaria saturada, la interrupción del acceso a servicios esenciales y la precariedad de los albergues improvisados configuran un entorno propicio para el uso empírico e indiscriminado de antimicrobianos y el resurgimiento inminente de brotes de infecciones inmunoprevenibles.

La evaluación de los riesgos de brotes infecciosos postdesastre debe interpretarse en el contexto de una crisis humanitaria preexistente en Venezuela, que ya había debilitado gravemente el programa nacional de inmunizaciones. Como antecedente crítico, el sistema sanitario nacional enfrentaba un deterioro significativo por limitaciones financieras y operativas causadas por factores socioeconómicos y políticos ^2^. La suspensión desde el año 2018 de vacunas esenciales contra el neumococo (PCV) y el rotavirus (RV) viene acumulando una cohorte infantil desprotegida. Sobre esta fragilidad basal, el sismo interrumpió el suministro eléctrico y de agua potable, inhabilitando la cadena de frío para la conservación de productos biológicos disponibles ^1^^-^^3^. Las coberturas vacunales ya eran bajas antes de la catástrofe, registrándose apenas un 51,1 % para la segunda dosis de sarampión (MCV2) y un 62,1 % para la vacuna pentavalente (DPT3) ^3^^,^^4^. Estas brechas de cobertura implican un riesgo inminente de transmisión activa y brotes epidémicos de enfermedades como el sarampión, las neumonías bacterianas y las diarreas agudas, especialmente en los refugios temporales, donde el hacinamiento acelera la propagación de estas patologías infecciosas ^4^^,^^5^. En tal sentido, distintos actores científicos y sanitarios han insistido en reforzar la vacunación como parte de la respuesta de emergencia, particularmente entre la población infantil y las personas desplazadas ^1^^,^^3^^-^^5^.

Conforme a las directrices de la Organización Mundial de la Salud (OMS), la respuesta no debe enfocarse en campañas de vacunación masivas, sino en restablecer con prontitud los esquemas de rutina ya instaurados ^(^^6^. Asimismo, se debe inmunizar a grupos de riesgo específicos como un componente preventivo de la respuesta inmediata. Esta prioridad técnica evita que las brechas basales de cobertura inmunitaria desencadenen brotes epidémicos de infecciones inmunoprevenibles en entornos de hacinamiento ^4^^-^^6^.

Paralelamente, las catástrofes naturales actúan como catalizadores indirectos para la emergencia y propagación de la resistencia antimicrobiana (RAM), un fenómeno bajo activa investigación en eventos telúricos recientes como los ocurridos en Turquía y Siria en 2023 ^7^. En situaciones de desastre, la elevada presión asistencial, la incertidumbre diagnóstica y la preocupación clínica, propician el uso empírico e indiscriminado de antimicrobianos de amplio espectro. Este empirismo ciego conduce a la administración inadecuada de antibióticos que ejercen una fuerte presión selectiva sobre la microbiota de los pacientes, induciendo una disbiosis. Además, fenómenos recurrentes como la suspensión de tratamientos, los cambios no planificados en los esquemas terapéuticos y la falta de seguimiento sistemático de las prescripciones, sustentan la hipótesis de que la propia respuesta a la emergencia puede transformarse en un potenciador de la crisis biológica en entornos hospitalarios y comunitarios ^7^^,^^8^.

Antes del doble terremoto, Venezuela ya registraba una carga significativa de RAM, sin una vigilancia epidemiológica integral, sin plan de acción nacional, con un sistema de salud fragmentado y la desigual distribución de recursos diagnósticos y terapéuticos ^3^^,^^9^. Al superponerse la emergencia telúrica a estas fragilidades basales, aumenta el riesgo de diseminación de infecciones asociadas a la atención de la salud (IAAS) en los centros asistenciales sobrecargados.

En este contexto de crisis, la microbiología clínica y la infectología desempeñan un papel estratégico. Los laboratorios de microbiología clínica son fundamentales para identificar patógenos en heridas traumáticas, neumonías y bacteriemias. Asimismo, proporcionan los perfiles de susceptibilidad antimicrobiana que orientan el tratamiento empírico inicial y su desescalada o reajuste ^(^^1^^,^^3^^,^^7^^,^^8^. La infectología, por su parte, permite elaborar protocolos adaptados a contextos de recursos limitados y asesorar en el uso racional de antimicrobianos. La experiencia internacional en terremotos recientes demuestra que la integración de la infectología, la microbiología y la vigilancia sanitaria en los comités de respuesta a desastres es fundamental ^7^^-^^9^. Esta articulación permite optimizar el diagnóstico y contener las infecciones por microorganismos multirresistentes, racionalizar el uso de los antibióticos y viabilizar las medidas de control adaptadas a la realidad de la emergencia.

Frente a este escenario, se requiere trascender desde la simple constatación de las vulnerabilidades hacia la formulación de una agenda de acción eficaz a partir de tres ejes estratégicos: En primer lugar, los planes de respuesta a desastres deben incorporar explícitamente la contención de la RAM y la prevención de las IAAS. Este eje estratégico se fundamenta en la evidencia epidemiológica que demuestra que los grandes terremotos inducen una «segunda ola» o emergencia sanitaria, caracterizada por heridas complejas por aplastamiento y altamente contaminadas, que requieren desbridamientos quirúrgicos múltiples y estancia prolongada en unidades de cuidados intensivos (UCI). Bajo la elevada presión asistencial en la fase de respuesta, estas condiciones clínicas aceleran la selección y dispersión de bacilos Gram negativos multirresistentes, tales como *Acinetobacter baumannii*, *Pseudomonas aeruginosa* y Enterobacterales productores de betalactamasas de espectro extendido (BLEE) y/o carbapenemasas, incrementando drásticamente las tasas de amputación, fallo orgánico y mortalidad postraumática ^7^^-^^9^. Considerando el entorno de infraestructura colapsada se debe priorizar el diagnóstico microbiológico, mediante estrategias de autonomía tecnológica. Tecnologías como la telemicrobiología y el uso de pruebas de diagnóstico rápido, incluidas las moleculares en el punto de atención (POCT, *point-of-care-testing*) permiten identificar patógenos y biomarcadores de resistencia desde las primeras horas ^9^^,^^10^. Esto permite abandonar el empirismo ciego, facilitar terapias dirigidas con antibióticos de espectro estrecho y preservar la utilidad clínica de los antimicrobianos de reserva frente a la selección de patógenos multirresistentes.

En segundo lugar, es fundamental implementar indicadores mínimos de vigilancia de la RAM y del consumo de antimicrobianos postdesastre. El monitoreo de las prescripciones en los entornos afectados debe llevarse a cabo conforme a las directrices de la Organización Panamericana de la Salud (OPS) y estructurarse bajo la clasificación AWaRe (del inglés *Access, Watch, Reserve*; Acceso, Precaución y Reserva) establecida por la OMS. Esta herramienta permite categorizar y auditar de forma ágil el uso de los recursos terapéuticos disponibles, priorizando la prescripción de antibióticos del grupo de Acceso y regulando de manera estricta la utilización empírica de los agentes de los grupos de Precaución y de Reserva por parte de los equipos médicos de respuesta ^11^. De este modo, la recolección simplificada de estos datos actúa como un mecanismo de alerta temprana ante desviaciones de los protocolos clínicos locales, y provee una métrica estandarizada y comparable para evaluar la calidad de la respuesta sanitaria en situaciones de crisis.

En tercer lugar, como pilar de la seguridad sanitaria, la respuesta de emergencia debe consolidar un eje de inmunizaciones que posicione a la vacunación como un componente operativo inmediato y no diferido ^4^^,^^6^. Las intervenciones deben estructurarse de manera multidimensional: desde el punto de vista de la inmunización clínico-asistencial actualizada, mediante la evaluación rápida del estado vacunal y la implementación de estrategias de inmunización integradas en la atención primaria y en los refugios, priorizando a la población infantil frente al sarampión (SRP), la difteria y la tos ferina (vacuna pentavalente), así como la vacunación contra la influenza y la COVID-19 en grupos de riesgo ^3^^,^^4^. En el ámbito del soporte infraestructural, es fundamental garantizar el restablecimiento sostenido de la inmunización de rutina, lo que implica asegurar el suministro de los productos biológicos, preservar la cadena de frío mediante generadores de emergencia y fortalecer la capacitación técnica del personal de salud ^3^. Finalmente, en la dimensión epidemiológica preventiva, la vacunación debe adaptarse al riesgo postdesastre, con medidas que incluyan la profilaxis antitetánica en heridas por escombros y la vacunación selectiva contra la hepatitis A ante la persistencia de condiciones de saneamiento deficientes en los albergues, en estrecha articulación con una vigilancia sindrómica activa ^3^^,^^4^.

La devastación causada por el doble terremoto en Venezuela constituye un recordatorio de que la salud pública no se construye únicamente sobre la gestión de la estabilidad cotidiana, sino sobre la capacidad de los sistemas sanitarios para anticipar, resistir y aprender de las crisis. Esta experiencia trasciende el ámbito nacional y debe proyectarse como un referente de abordaje para toda Latinoamérica, con especial énfasis en la franja andina. Este marco referencial permite capitalizar las intervenciones exitosas y evaluar críticamente las fallas operativas, transformando las lecciones aprendidas en una oportunidad para mejorar la resiliencia institucional ante futuras emergencias. Esta región comparte una elevada vulnerabilidad geofísica, causada por los complejos sistemas tectónicos activos, lo que la expone de forma recurrente a desastres sísmicos de gran magnitud. A esta fragilidad estructural se suma una susceptibilidad climática extrema ante fenómenos meteorológicos como El Niño, cuyas alteraciones pluviométricas y térmicas favorecen la proliferación de vectores y exacerban las epidemias en contextos de hacinamiento y desventaja social. Cuando tales catástrofes impactan en sistemas sanitarios previamente debilitados, caracterizados por coberturas de inmunización subóptimas y el colapso de los servicios de agua, saneamiento e higiene, las consecuencias sanitarias rebasan el trauma agudo inmediato. Salvaguardar el acceso a las vacunas y fortalecer la inmunidad colectiva en situaciones de crisis constituye un componente operativo de primera línea esencial para contener brotes previsibles. Paralelamente, en esta delicada intersección, la resistencia antimicrobiana (RAM) se configura como una segunda crisis biológica superpuesta y silenciosa. Por consiguiente, es ineludible que las políticas de gestión de desastres en la región incorporen la contención de la RAM, la relevancia del diagnóstico microbiológico y la preservación de los programas de inmunización como pilares fundamentales de la seguridad sanitaria colectiva.
